# An Egr-1-specific DNAzyme regulates Egr-1 and proliferating cell nuclear antigen expression in rat vascular smooth muscle cells

**DOI:** 10.3892/etm.2013.1013

**Published:** 2013-03-15

**Authors:** JUNBIAO ZHANG, CHANGLEI GUO, RAN WANG, LULI HUANG, WANQIAN LIANG, RUNNAN LIU, BING SUN

**Affiliations:** 1 Departments of Cardiovascular Internal Medicine, The First Affiliated Hospital of Xinxiang Medical University, Weihui, Henan 453100;; 2 Hematology, The First Affiliated Hospital of Xinxiang Medical University, Weihui, Henan 453100;; 3 Department of Cardiovascular Internal Medicine, The First Affiliated Hospital, China Medical University, Shenyang, Liaoning 110001;; 4 Department of Tuberculosis, The First Affiliated Hospital of Xinxiang Medical University, Weihui, Henan 453100, P.R. China

**Keywords:** serum response factor, DNA, catalytic, muscle cells, smooth muscle, proliferating cell nuclear antigen

## Abstract

The aim of the present study was to transfect rat aortic smooth muscle cells with an early growth response factor-1 (Egr-1)-specific DNAzyme (ED5), to observe its effect on Egr-1 and proliferating cell nuclear antigen (PCNA) expression and to elucidate the mechanism of ED5-mediated inhibition of vascular smooth muscle cell (VSMC) proliferation. VSMCs in primary culture obtained by tissue block adhesion were identified by morphological observation and α smooth muscle actin (α-SM-actin) immunocytochemistry. The cells were then transfected with ED5 or scrambled ED5 (ED5SCR). The three groups of cells used in the present study were the control group, ED5 group and ED5SCR group. The expression levels of Egr-1 and PCNA protein were detected following transfection by analyzing and calculating the integral optical density value in each group. Primary culture of VSMCs and transfection of ED5 and ED5SCR were successfully accomplished. Following stimulation with 10% fetal calf serum, the Egr-1 protein was expressed most strongly at 1 h and demonstrated a declining trend over time; the expression of PCNA protein began at 4 h, peaked at 24 h and then demonstrated a slightly declining trend over time. Compared with the control group and the ED5SCR group, ED5 inhibited the expression of Egr-1 and PCNA (P<0.05). ED5 was able to inhibit the expression of Egr-1 and PCNA proteins in VSMCs to a certain extent and VSMC proliferation *in vitro*. DNAzyme gene therapy may be useful as a new method for treating vascular proliferative diseases, including atherosclerosis and restenosis.

## Introduction

Restenosis (RS) following percutaneous coronary intervention (PCI) is a type of repair response to local vascular injury and is a local vascular reconstruction comprehensively mediated by various cytokines (1,2). Vascular smooth muscle cell (VSMC) proliferation is a complex biological process finely controlled by various cytokines (3). Early growth response factor-1 (Egr-1), a member of the immediate-early gene family, is an important nuclear transcription factor that regulates the expression of various cell proliferation-related genes and promotes cell proliferation and migration (4). Deoxyribozymes (DNA enzymes, DRz) are deoxyribose molecules with enzyme activity. Due to their phosphoesterase activity, DRz are able to catalyze the splicing of specific RNA (5) and may be used as an effective treatment tool for various stages of a disease. The present study elucidates the mechanism by which an Egr-1-specific DNAzyme (10–23 DNA enzyme, ED5) inhibits smooth muscle cell proliferation by observing, at the cellular and molecular levels, the effect of ED5 on the expression of Egr-1 and PCNA following the transfection of VSMCs. This study also proposes a novel gene therapy technique for the prevention of RS following PCI.

## Materials and methods

### Materials

Healthy Wistar rats, aged 5–6 years and weighing 120–150 g, were used in the present study. ED5 and ED5SCR were synthesized by Takara Biotechnology (Dalian) Co., Ltd. (China). The ED5 sequence was 5′-CCGCTGCCAGGCTAGC TACAACGACCCGGACGT-3′ and the ED5SCR sequence was 5′-GCCAGCCGCGGCTAGCTACAACGATGGCTCCAC-3′. The two sequences were purified by polyacrylamide gel electrophoresis, lyophilized and recovered. The 5′ and 3′ ends were sodium-modified and the 5′ ends of certain ED5 molecules were labeled with fluorescein isothiocynate (FITC).

### Cell culture and identification

The rat thoracic aorta was quickly removed under aseptic conditions following intraperitoneal injection of 10% chloral hydrate and immediately rinsed with aseptic phosphate-buffered saline (PBS) containing penicillin (100 g/ml) and streptomycin (100 g/ml). After stripping the extravascular connective tissue and epineurium, the vessels were longitudinally cut off and the internal membrane was positioned tilted upward in the glass culture dish. Trypsin (0.25%) was smeared onto the endometrium for 1 min and then the digestion was terminated with a medium containing 20% fetal calf serum (FCS) to achieve a transparent, thinner and tougher mesosome. The mesosome was cut into 1 mm^2^ sized tissue blocks. The endometrial sections were positioned tilted upward in the plastic culture dish and separated from each other by 5 mm. These tissue blocks were left to stand at 37°C in a 5% CO_2_ saturated humidity incubator for 1–2 h and submerged in 2 ml medium after firm adhesion. After 5–7 days, 10% FCS medium was used to extract the cells from the tissue blocks. Third to fifth generation cells were selected for the experiments. This study was conducted in accordance with the declaration of Helsinki. This study was conducted with approval from the Ethics Committee of The First Affiliated Hospital of Xinxiang Medical University (Weihui, China).

### Cell identification

Immunocytochemical identification of α-smooth muscle anti-actin was performed. The morphology of the cells was observed under an inverted phase-contrast microscope. Cells were streptavidin-peroxidase labeled with horseradish peroxidase, with positive staining shown by a tan/yellow cytoplast.

### Experimental groups

Three groups were used in the study: the control group, the ED5 group and the ED5SCR group. Different sub-groups according to various monitoring indicators and transfection time were also employed.

### Transfection

Serum- and antibiotic-free Dulbecco’s modified Eagle’s medium (DMEM) was placed in a sterile Eppendorf tube, mixed with an additional quantity of FuGENE6 and then incubated at room temperature for 5 min. FuGENE6 and oligonucleotides (ED5, ED5SCR) were mixed at 3:1 (volume: mass), with incubation at room temperature for 15 min. Upon reaching 70% fusion, the VSMCs were cultured for 30 h in serum- and antibiotic-free DMEM and then transfected with 0.1 *μ*mol/l transfection complex. After 18 h, 10% FCS and antibiotic-free DMEM were exchanged for the second transfection. Continuous culture for 1–3 days was performed for monitoring indicators. Fluorescence microscopy revealed that the transfected cells displayed the yellow-green fluorescence of FITC.

### Immunocytochemistry and western blot analysis

The cell slides were created at 4°C and fixed with 75% alcohol for 30 min prior to storing at −20°C. Following the instructions of the streptavidin-peroxidase kit, rabbit polyclonal anti-rat Egr-1 antibody (1:100 dilution) and PCNA antibody (1:200 dilution) were used. For 3,3′-diaminobenzidine (DAB) staining, a tan-yellow cytoplast or nucleus indicated positive staining. The cell slides were counterstained with hematoxylin, differentiated with hydro-chloric acid alcohol, dehydrated with graded ethanol, vitrified with dimethylbenzene and sealed with gum. The integral optical density value was analyzed and calculated by a MetaMorph image analysis system (three pieces were removed from each group and five different fields of view were freely selected for each piece). A total of 50 *μ*g total extracted protein was separated by polyacrylamide gel electrophoresis, transferred and blocked. Egr-1 polyclonal antibody (Santa Cruz Biotechnology Inc., Santa Cruz, CA, USA; 1:500) and PCNA monoclonal antibody (Boster Biological Technology Ltd., Fremont, CA, USA; 1:500) were individually added for overnight incubation. Incubation was then repeated with secondary antibodies labeled with horseradish peroxidase and dyed for observation.

### Statistical analysis

SPSS 13.0 statistical software (SPSS Inc., Chicago, IL, USA) was used to perform statistical analyses, with mean ± standard deviation as the measurement data. Single factor analysis of variance was used to compare the means among the groups. P<0.05 was considered to indicate a statistically significant difference.

## Results

### VSMC identification

**Figure 1:**
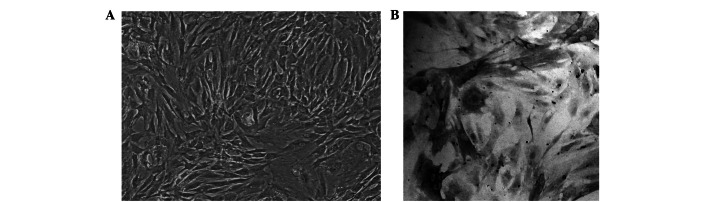
VSMC primary culture and identification. (A) VSMC ‘peak-to-valley’ growth (magnification, 100×); (B) VSMC α-SM-actin immunocytochemical staining (S-P method; magnification, 400×). VSMC, vascular smooth muscle cell; SM, smooth muscle.

Morphological observation revealed that after primary culture for 5–6 days, long spindle-shaped cells with strong cytoplasmic refractivity, oval nucleus and rich cytoplasm migrated out from the tissues. Part of these cells overlapped, forming typical VSMC ‘peak-to-valley’ growth (Fig. 1A) (6). Alpha smooth muscle actin (α-SM-actin) immunocytochemical staining revealed an abundance of tan-yellow myonemes in the cytoplasm, which were aligned parallel to the vertical axis of the cells. The nuclei were light blue following counterstaining with hematoxylin, proving that the cells obtained were VSMCs with high purity (Fig. 1B).

### Transfection efficiency and cell survival rate

After being transfected twice, the cells presented the yellow-green fluorescence of FITC when observed by fluorescence microscopy. The transfection efficiencies of ED5 (70±1.25%) and ED5SCR (72±1.63%) were high, according to the statistical analyses. The VSMCs were continually cultured for 72 h following transfection, without the presence of numerous apoptotic or necrotic cells, and achieved a cell survival rate of >99%.

### Egr-1 protein expression

**Figure 2:**
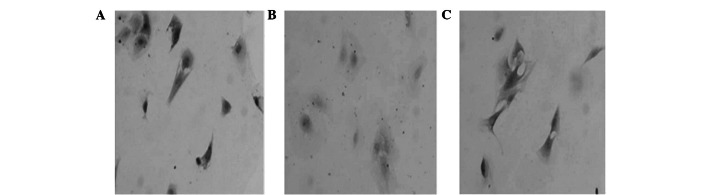
Egr-1 protein expression in each group after ED5 transfection for 1 h (magnification, 100×) in the (A) control, (B) ED5 and (C) ED5SCR groups. Egr-1, early growth response factor 1; ED5, Egr-1-specific DNAzyme; ED5SCR, scrambled ED5.

Cell chemical staining and western blot analysis revealed that Egr-1 protein in the three groups was expressed most strongly 1 h after serum stimulation (Fig. 2); however, a declining trend was observed over time (Table I). Egr-1 protein expression in the ED5 group was inhibited and this inhibition was statistically significant compared with the two control groups at four different time points.

### PCNA expression

**Figure 3:**
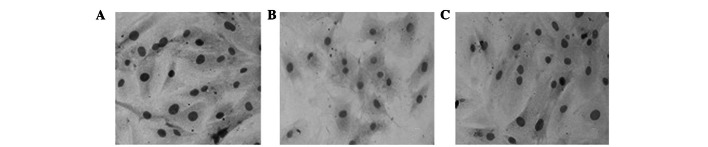
PCNA protein expression in each group after transfection for 24 h (magnification, ×400) in the (A) control, (B) ED5 and (C) ED5SCR groups. PCNA, proliferating cell nuclear antigen; ED5, Egr-1-specific DNAzyme; Egr-1, early growth response factor 1; ED5SCR, ED5 and scrambled ED5.

Cell chemical staining and western blot analysis revealed that the expression of PCNA protein in all three groups began at 4 h, peaked at 24 h, and thereafter demonstrated a slightly declining trend over time (Fig. 3 and Table II). The level of PCNA protein expression in the ED5 group was inhibited to a certain extent at the four different time points.

## Discussion

Over the last 20 years, coronary intervention treatment has made tremendous progress. Stent implantation has been used in a wide range of applications and is now applied in >70% of all coronary intervention treatments (7). The RS rate has been significantly reduced. In a clinical trial, rapamycin-coated stents demonstrated good effects in preventing RS (8). However, the RS and progressive thrombogenesis may occur after drug stenting (9). Currently, the mechanism of RS stenting causes endothelial injury, platelet activation, VSMC proliferation and migration, increased extracellular matrix and neointimal hyperplasia (10). It has been reported that VSMC proliferation and migration are the main causes of RS occurrence (11). Given previous developments in molecular biology, gene therapies inhibiting smooth muscle cell proliferation have become important for RS treatment.

Egr-1, a zinc finger transcription factor, is expressed at low levels or not expressed at all in normal vessel walls. Egr-1 induces VSMC and endothelial cell expression under arterial injury and other stimulation, promoting VSMC proliferation and endometrial thickening (12). Previous studies have shown that Egr-1 antisense oligonucleotides or the ‘lure strategy’ successfully suppresses Egr-1 expression following arterial injury and VSMC proliferation (13,14). Moreover, in a rat carotid artery balloon injury model, ED5 restrained Egr-1 expression and suppressed VSMC proliferation and internal membrane thickening (14,15), which stopped the occurrence and development of RS to a certain extent. However, the specific mechanism involved in this biological function remains unclear.

The expression of PCNA, a nuclear peptide synthesized or expressed only in proliferating cells, begins to increase in the late G1 phase and then reaches a peak in the S phase, and is an important index of cell proliferation. Its expression level is directly proportional to the degree of cell proliferation; thus, PCNA detection is a reliable index by which to evaluate the cell proliferation state (16,17). Inhibiting the core process of cell proliferation/cell cycle by intervening in the expression of cell cycle-associated proteins is an effective method of preventing RS (18). We consider that ED5 plays a role in these processes by regulating PCNA and Egr-1 expression.

The present study demonstrates that Egr-1 promotes VSMC proliferation. ED5 inhibits Egr-1 and PCNA protein expression to a certain extent following transfection of VSMCs cultured *in vitro*. ED5 also inhibits the proliferation of VSMCs cultured *in vitro*, an observation that may lead to a new method of gene therapy that prevents postoperative RS following PCI. Currently, RS gene therapy has shown good treatment effects by regulating various target genes in animal experiments. However, its successful application in the human body has not yet been reported. Whether or not treatment of a single target gene is effective in the complex human body remains to be studied.

This study further demonstrates that Egr-1 is an important nuclear transcription factor that promotes VSMC proliferation. ED5 was shown to inhibit the proliferation of VSMCs cultured *in vitro* by reducing Egr-1 and PCNA protein expression at the cellular and molecular levels. The findings of this work provide a new method of gene therapy for the prevention and treatment of RS.

## Figures and Tables

**Table I:** Optical density change of Egr-1 in each group at various time points.

Group	1 h	4 h	24 h	48 h	72 h
Control	44.15±4.21	31.71±1.90	22.18±1.35	13.48±0.73	11.09±0.76
ED5	29.23±2.13^a^	20.27±2.09^a^	14.37±1.37^a^	8.46±0.86^a^	10.27±0.21
ED5SCR	44.54±3.37	33.04±2.50	22.06±2.20	13.58±1.09	10.45±1.30

Data are presented as mean ± standard deviation.

a P<0.05, vs. the control and ED5SCR groups. Egr-1, early growth response factor 1; ED5, Egr-1-specific DNAzyme; ED5SCR, scrambled ED5.

**Table II:** Optical density change of PCNA in each group at various time points.

Group	4 h	24 h	48 h	72 h
Control	11.61±1.09	19.13±2.84	15.64±1.12	13.94±1.82
ED5	6.20±1.33^a^	6.22±0.24^a^	7.87±0.79^a^	5.79±0.64^a^
ED5SCR	12.12±1.43	18.74±2.20	15.73±0.63	14.28±1.35

Data are presented as mean ± standard deviation.

a P<0.05, vs. the control and ED5SCR groups. PCNA, proliferating cell nuclear antigen; ED5, Egr-1-specific DNAzyme; Egr-1, early growth response factor 1; ED5SCR, scrambled ED5.
